# Interplay between Oxidative Stress and Nutrient Sensing Signaling in the Developmental Origins of Cardiovascular Disease

**DOI:** 10.3390/ijms18040841

**Published:** 2017-04-15

**Authors:** You-Lin Tain, Chien-Ning Hsu

**Affiliations:** 1 Department of Pediatrics, Kaohsiung Chang Gung Memorial Hospital and Chang Gung University College of Medicine, Kaohsiung 833, Taiwan; tainyl@hotmail.com; 2 Institute for Translational Research in Biomedicine, Kaohsiung Chang Gung Memorial Hospital and Chang Gung University College of Medicine, Kaohsiung 833, Taiwan; 3 Department of Pharmacy, Kaohsiung Chang Gung Memorial Hospital, Kaohsiung 833, Taiwan; 4 School of Pharmacy, Kaohsiung Medical University, Kaohsiung 807, Taiwan

**Keywords:** arginine, asymmetric dimethylarginine, cardiovascular disease, developmental origins of health and disease (DOHaD), hypertension, nutrient sensing, nitric oxide, oxidative stress, phytonutrient, symmetric dimethylarginine

## Abstract

Cardiovascular disease (CVD) presents a global health burden, despite recent advances in management. CVD can originate from early life by so-called “developmental origins of health and disease” (DOHaD). Epidemiological and experimental evidence supports that early-life insults can induce programming of later CVD. Underlying the DOHaD concept, early intervention may offset programming process to prevent the development of CVD, namely reprogramming. Oxidative stress and nutrient sensing signals have been considered to be major mechanisms of cardiovascular programming, while the interplay between these two mechanisms have not been examined in detail. This review summarizes current evidence that supports the link between oxidative stress and nutrient sensing signaling to cardiovascular programming, with an emphasis on the l-arginine–asymmetric dimethylarginine (ADMA)–nitric oxide (NO) pathway. This review provides an overview of evidence from human studies supporting fetal programming of CVD, insight from animal models of cardiovascular programming and oxidative stress, impact of the l-arginine–ADMA–NO pathway in cardiovascular programming, the crosstalk between l-arginine metabolism and nutrient sensing signals, and application of reprogramming interventions to prevent the programming of CVD. A greater understanding of the mechanisms underlying cardiovascular programming is essential to developing early reprogramming interventions to combat the globally growing epidemic of CVD.

## 1. Introduction

Cardiovascular disease (CVD) remains a crucial challenge for public health. Despite advances in medical and surgical treatment, CVD still accounts for most noncommunicable disease (NCD) deaths all over the world [1]. CVD can originate from early life, referred to as the developmental origins of health and disease (DOHaD) [2]. A growing body of epidemiological and experimental evidence supports that adverse environments on pregnancy (including nutrition) lead to permanent alterations of function and structure in specific organs that are vulnerable to develop CVD in later life, namely cardiovascular programming [3,4,5]. On the other hand, the DOHaD concept opens a new window in a way that helps to offset the programming process in early life in order to prevent the development of CVD in the later lifespan, through so-called reprogramming [3,6].

So far, a number of hypothetical mechanisms, including oxidative stress [7,8], dysregulation of nutrient-sensing signals [9], activation of renin-angiotensin system (RAS) [10], low nephron endowment [11], glucocorticoid effect [12], epigenetic regulation [13], and sex differences [4,5], have been examined in relation to cardiovascular programming and its comorbid illness [3,4,5]. For details, please refer to previous reviews [3,4,5,6,7,8,9,10,11,12,13]. Although oxidative stress and nutrient sensing signals have been extensively studied individually, the emerging link between these two mechanisms in cardiovascular programming have not been studied in detail.

Oxidative stress is an oxidative shift characterized by an imbalance between pro-oxidant molecules and antioxidant defenses, mainly related to dysregulation of reactive oxygen species (ROS) and nitric oxide (NO). Although ROS are by-products of physiological respiration, they are major players in the pathogenesis of all sorts of diseases. NO is a free radical that is considered to be toxic or protective depending on its concentration, subcellular localization, and reaction with ROS. Oxidative stress is frequently associated with in utero programming in a number of pregnancy-associated diseases such as pregnancy-induced hypertension, preeclampsia, gestational diabetes, malnutrition, premature labor, preterm prelabor rupture of the membranes, intrauterine growth restriction, maternal stress, and placental insufficiency [5,8,14]. Moreover, diseases originating in the perinatal and neonatal periods such as bronchopulmonary dysplasia, retinopathy of prematurity, necrotizing enterocolitis, and periventricular leukomalacia are closely related to oxidative stress [15]. Newborns, especially if preterm, are particularly vulnerable to oxidative stress because they exhibit accelerated production of ROS and limited antioxidant protection, which increases susceptibility to disease later in life. Oxidative stress-mediated programming may act directly through epigenetic regulation of gene expression or indirectly via the effects of certain free radical signals [5,8,14]. However, which particular ROS or redox-sensitive signaling is responsible for cardiovascular programming remains unclear. So far, there remains a lack of data on how and when to reprogram oxidative stress-related programmed diseases [16].

Works that haven been published in recent years support that NO–ROS imbalance is important for the developmental programming of CVD [4,5,6,7,8]. Among the reasons for NO–ROS imbalance, increasing attention has been centered on asymmetric dimethylarginine (ADMA) [17]. ADMA can compete with l-arginine (the substrate for NO synthase) to reduce the synthesis of NO, but on the other hand, it induces superoxide production by uncoupling nitric oxide synthase (NOS). Thus, cellular ADMA concentrations tightly regulate the local NO–ROS balance [17,18,19]. A growing body of evidence from clinical trials and animal studies suggest an impact of ADMA related NO–ROS imbalance on the developmental programming of CVD and cardiovascular outcome [14,19,20,21,22].

Next, the important role of l-arginine in normal pregnancy, fetal growth and development, and cardiovascular health has been reviewed elsewhere [23,24,25,26]. l-arginine and other amino acids, sugars, and fatty acids are important cellular nutrients. Specific nutrients have been recognized as signaling molecules transmitting and translating dietary signals into changes in gene expression via the appropriate sensing mechanisms, also known as nutrient sensing signaling pathway [27]. Increasing evidence supports that the l-arginine–NO pathway regulates the metabolism of energy substrates and nutrient sensing signals [28].

**Figure 1:**
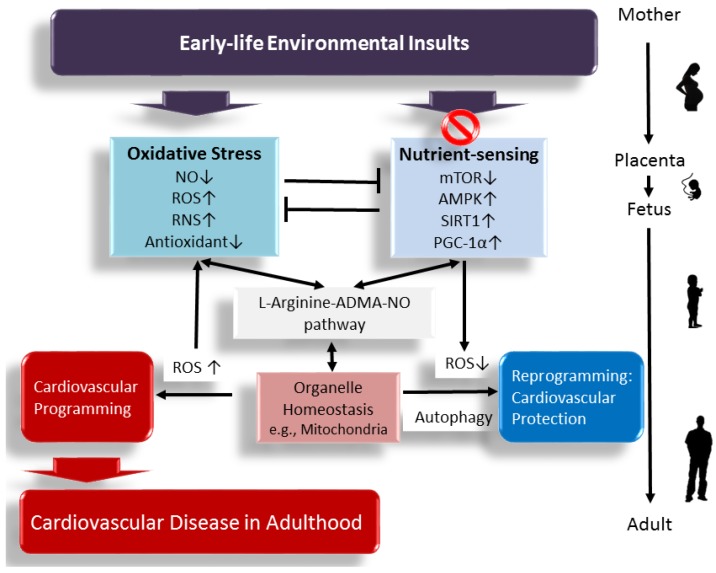
A schema showing that early-life environmental insults affect the l-arginine–ADMA–NO pathway, increase oxidative stress, and dysregulate nutrient sensing signals, leading to cardiovascular programming and cardiovascular disease (CVD) in later life. Early targeting of the above mechanisms might serve as a reprogramming approach to prevent CVD and its related comorbidities in adulthood. ↑ = increased. ↓ = decreased.

This review aims to summarize evidence linking the l-arginine–ADMA–NO pathway to the development of CVD, with a focus on the interplay between oxidative stress and nutrient sensing signaling, and provide various early manipulations targeting the above mechanisms as a reprogramming approach to prevent the cardiovascular programming and its related comorbidities across the later lifespan. A schematic summarizing the links between the l-arginine–ADMA–NO pathway, oxidative stress, nutrient sensing signals, and cardiovascular programming is presented in Figure 1.

## 2. Evidence for Programming of Cardiovascular Disease in the Human

Important support for developmental origins of CVD came from observations on the severe famines of 1944–1945 in The Netherlands [29], of 1941–1944 in Saint Petersburg [30], and of 1967–1971 in the Biafra [31]. Offspring exposed to famine during pregnancy are prone to develop a number of chronic diseases, including hypertension and coronary artery disease [29,30,31,32,33,34]. Another line of evidence comes from studies of twin pregnancies. In twins, there was a positive association between birth weight and blood pressure (BP) in infants [35]. The lighter twins are prone to die from heart disease [36]. However, it is practically impossible to test prospectively for critical windows in human development, and to gather such information from most epidemiological studies.

As reviewed elsewhere [6], the risk of programmed hypertension has been assessed in a number of mother-child cohorts. Several risks affecting early-life BP of offspring in these cohorts include undernutrition, smoking, low vitamin D intake, gestational hypertension, maternal obesity, short-term breastfeeding, and excessive postnatal weight gain [6]. However, these cohorts cannot yet per se directly establish a causal relationship between the specific insult and phenotypes of CVD. Some DOHaD theories have been developed to explain these epidemiological observations, such as thrifty phenotype [37], catch-up growth hypothesis [38], and predictive adaptive responses [39]. Nevertheless, these hypotheses do not advocate molecular mechanisms by which way the phenotype is generated. As a consequence of ethical considerations concerning what is feasible or not in human studies, animal models are of critical importance. It stands to reason that much of our knowledge seems to come mainly from animal models, which identify how types of early life insults may program cardiovascular phenotypes, which developmental window is critical for cardiovascular programming, and what reprogramming strategy can be used.

## 3. Impact of Oxidative Stress on Cardiovascular Programming in Animal Models

Mounting evidence from animal studies confirms the link between adverse conditions in early life, cardiovascular programming, and oxidative stress. The most common species used in DOHaD field are rodents, as well as rabbits, sheep, pigs, and non-human primates and will depend on their similarity to the human being like developmental window, length of gestation, placental structure, and litter size [40,41,42]. Since cardiovascular phenotypes often develop after a prolonged asymptomatic phase in childhood and for the sake of brevity, we have restricted this review to data obtained from adult offspring. Here we summarize in Table 1 studies documenting cardiovascular programming related to oxidative stress [43,44,45,46,47,48,49,50,51,52,53,54,55,56,57,58,59,60,61,62,63,64,65,66,67,68].

A number of early-life insults have been reported to cause cardiovascular programming related to oxidative stress, including undernutrition [43,48,51,54,55,61,63,64], streptozotocin (STZ)-induced diabetes [44,62], preeclampsia [45,46,47], prenatal hypoxia [49,56], maternal nicotine exposure [59,60], ethanol consumption [50], maternal inflammation [57], prenatal glucocorticoid exposure [52,53,65,67,68], and maternal high-fat intake [58,66]. Limited information is available using large animals to study oxidative stress and cardiovascular programming simultaneously [67,68]. Despite early-life insults induced cardiovascular outcome and oxidative stress having been reported individually in young nonhuman primate offspring [69,70], there remains a lack of data regarding their long-term interrelationship. As shown in Table 1, the rat is the most widely used laboratory mammal in this research field. Rats grow rapidly during their childhood and become sexually mature at approximately the sixth week. In adulthood, one rat month is comparable to three human years [42]. Female rats enter menopause between the ages of 15 and 20 months. Thus, Table 1 lists cardiovascular outcomes evaluated at different ages, which allows calculations to refer to humans of a specific age group.

Since embryo development occurs in a relatively low-oxygen environment [7], the developing fetus is highly vulnerable to oxidant injury. Oxidative stress can develop from birth through adulthood to old age in different experimental models of cardiovascular programming [7]. Insults need only last for a brief moment during pre- or peri-natal periods, as little as a few days [57], to cause long-term cardiovascular consequences. Also, early postnatal environment can influence redox balance to elicit cardiovascular programming. In an early postnatal overfeeding model [71], elevation in BP and cardiac hypertrophy are observed at weaning. In addition, impaired heart contractility, cardiac fibrosis, and increased vulnerability to ischemia-reperfusion injury have been observed in adulthood. These observations suggest that there are a number of critical windows during prenatal and postnatal life, and that a range of insults for cardiovascular programming should be taken into account. Studies listed in Table 1 indicate that cardiovascular programming can be attributed to multiple hits [49,53], although oxidative stress is unlikely to constitute the sole programmed mechanism that increases the vulnerability to later superimposed cardiovascular injury. Therefore, it is important to consider the potential interactions between the pre- and post-natal environment and between oxidative stress and other programmed mechanisms in determining final cardiovascular outcomes.

Although the common pathogenic mechanisms of cardiovascular programming are still inconclusive, animal models have provided certain mechanisms, including, but not limited to, renin-angiotensin system (RAS), endothelial dysfunction, epigenetics, glucocorticoid effects, low nephron endowment, oxidative stress, and dysregulated nutrient sensing signals [3,4,5,6,7,8,9,10,11,12,13]. Importantly, among these hypothetical mechanisms, the l-arginine–ADMA–NO pathway is closely interrelated to the others in determining the programming process.

## 4. Impact of ADMA Induced NO–ROS Imbalance in Cardiovascular Programming

**Figure 2:**
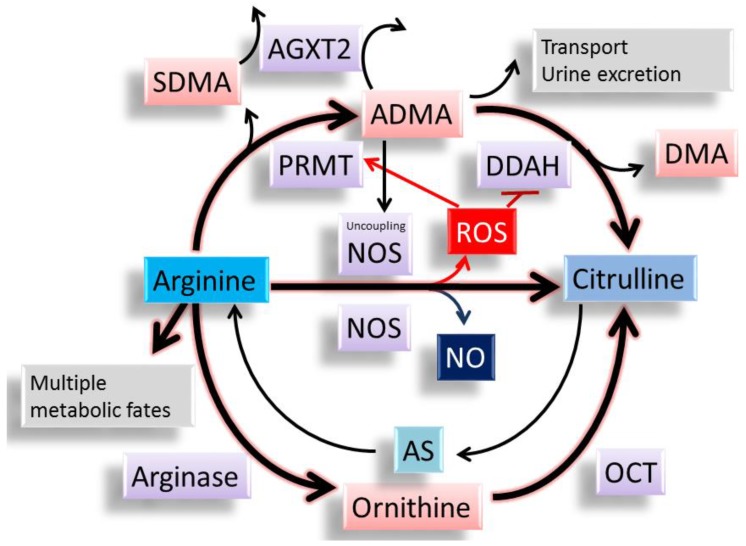
The synthesis and metabolism of l-arginine, asymmetric dimethylarginine (ADMA), and l-citrulline. l-arginine has multiple metabolic fates, including metabolism by NOS, arginase, and other enzymes. ADMA can compete with l-arginine to reduce the synthesis of NO. Both ADMA and symmetric dimethylarginine (SDMA) come from methylated l-arginine by protein arginine methyltransferase (PRMT). ADMA can be transported to other organs or excreted into the urine. Unlike SDMA, only ADMA can be metabolized by dimethylarginine dimethylaminohydrolase (DDAH)-1 and -2. Alanine-glyoxylate aminotransferase 2 (AGXT2) can metabolize ADMA as well as SDMA. l-citrulline can be generated by NOS, DDAHs, and ornithine carbamoyltransferase (OCT). l-citrulline can be used to make l-arginine via the argininosuccinate (AS) pathway. ADMA can uncouple NOS to produce reactive oxygen species (ROS). ROS can induce PRMT and inhibit DDAH activity, leading to an increase in ADMA.

Emerging evidence demonstrates that ADMA is involved in the development of CVD [19,20,21,22]. As we reviewed before [14], many studies support that ADMA related NO-ROS imbalance plays a major role in compromised pregnancy and fetal programming. ADMA is an l-arginine analogue, which can compete with l-arginine to inhibit the activity of NOS, resulting in the reduction of NO (Figure 2). Protein-incorporated ADMA is a methylated arginine derivative generated by the addition of methyl groups in arginine residue in proteins through the type I protein arginine methyltransferase (PRMT) family. Free ADMA is then released after protein degradation [17,18,19]. Free ADMA can be transported to other organs by cationic amino acid transporter or excreted into the urine. Nearly 80% of ADMA is metabolized by dimethylarginine dimethylaminohydrolase-1 (DDAH-1) and -2 (DDAH-2), and alanine-glyoxylate aminotransferase 2 (AGXT2) [17,18,19]. On the other hand, our body can use l-citrulline to make l-arginine via the argininosuccinate (AS) pathway. l-arginine can also be metabolized by arginase to generate ornithine, which can be further converted to l-arginine by ornithine carbamoyltransferase (OCT). Therefore, there is a close interplay between l-arginine and ADMA to control the synthesis and metabolism of each other. Another isomer of ADMA methylated by PRMTs are symmetric dimethylarginine (SDMA). Unlike enzymatically metabolized ADMA, SDMA is eliminated primarily by renal excretion. High plasma ADMA or SDMA concentrations not only predict all-cause mortality and CVD events, but are also relevant to a broad range of diseases [19].

ROS have been shown to increase PRMT and inhibit DDAH activity, leading to an increase in ADMA [72,73]. Further, high levels of ADMA can uncouple NOS isoenzymes to produce superoxide instead of NO, contributing to the burden of oxidative stress [74]. Thus, ADMA per se can lead to the production of ROS and reduction of NO. Indeed, increasing evidence indicates that ADMA-related NO–ROS imbalance is critical in the development of hypertension and CVD, as reviewed elsewhere [19,20,21,22].

Several lines of evidence indicate that the ADMA induced NO–ROS imbalance interacts with other mechanisms to incite cardiovascular programming. First, a growing body of evidence indicates that angiotensin II-induced increases of ADMA and oxidative stress contribute to the development of CVD and hypertension [75,76], whereas early blockade of the RAS has been shown to reduce ADMA and prevent the development of hypertension [77,78]. Second, ADMA competes with l-arginine to inhibit NOS activity and is involved in endothelial dysfunction [17,18]. It has been well established that endothelial dysfunction is central to the development of CVD [24]. Third, there are studies that show that redox signaling and NO control epigenetic regulation, contributing to cardiovascular programming [79]. Fourth, there are many reports that NO-ROS imbalance plays a crucial role in several models of glucocorticoid-induced programmed hypertension [52,53,80,81,82]. Last, increasing evidence supports that the l-arginine–NO pathway regulates the metabolism of energy substrates and nutrient sensing signals [28]. All of these observations provide a close link between the l-arginine–ADMA–NO pathway and other important mechanisms involved in cardiovascular programming.

## 5. Metabolic Crosstalk between Arginine Metabolism and Nutrient Sensing Signaling

**Figure 3:**
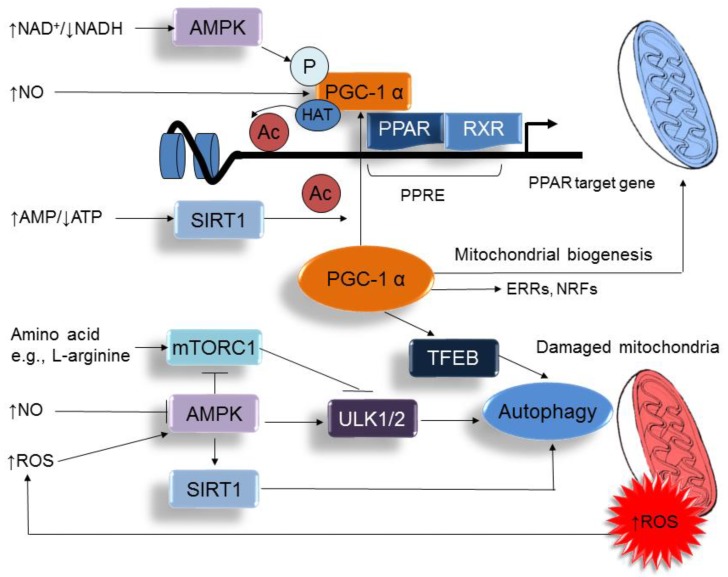
A schema showing the interplay between nutrient sensing signals and oxidative stress on the regulation of PPARγ coactivator-1α (PGC-1α), peroxisome proliferator-activated receptor (PPAR) target genes, mitochondria biogenesis, and autophagy. ↑ = increased. ↓ = decreased.

Maternal nutrition plays a critical role in placental function, fetal growth, organogenesis, and development. As shown in Table 1, a number of nutritional insults can induce cardiovascular programming [43,48,51,54,55,58,61,63,64,66]. Since altered maternal nutrition causes disturbed nutrient-sensing signals [9], nutrient-sensing signaling might therefore be a common mechanism underlying nutritional programming of CVD. Nutrient-sensing signaling pathways orchestrate fetal metabolism and development in response to maternal nutritional insults. Accordingly, a number of these signaling pathways exist in the cardiovascular system, including silent information regulator transcript (SIRT), cyclic adenosine monophosphate (AMP)-activated protein kinase (AMPK), peroxisome proliferator-activated receptors (PPARs), PPARγ coactivator-1α (PGC-1α), and mammalian target of rapamycin (mTOR) pathway. Among them, PGC-1α acts as a hub for a nutrient-sensing cluster. As shown in Figure 3, activation of AMPK by increased NAD^+^/NADH ratio, activation of SIRT1 by increased mitochondrial AMP/adenosine triphosphate (ATP) ratio, or NO can affect PGC-1α activity to promote mitochondria biogenesis [83,84]. AMPK induces mitochondrial biogenesis by activating PGC-1α either directly or through SIRT1 [83,85]. In addition, SIRT1 and AMPK can mediate deacetylation and phosphorylation of PGC-1α, respectively [85], to regulate the expression of PPAR target genes. Early-life nutritional insults might drive nutrient sensing signals to regulate PPARs and their target genes and thereby go through cardiovascular programming [86,87]. A recent review by us demonstrated that several PPAR target genes contribute to programmed hypertension, such as *Nos2*, *Nos3*, *Sod2*, *Nrf2*, *Sirt7*, *Ren*, and *Sgk1* [88]. Furthermore, our reports demonstrated that the PPAR signaling pathway is significantly regulated in a variety of models of programmed hypertension, such as maternal caloric restriction [89], STZ-induced maternal diabetes [89], prenatal dexamethasone exposure [81], and maternal high-fructose consumption [90]. In addition to PPARs, PGC-1α can activate other nuclear receptors, like estrogen receptor related receptor (ERR) and nuclear respiratory factor (NRF) to regulate energy metabolism [85].

PGC-1α not only interacts with nutrient sensing signals to regulate mitochondrial biogenesis, but also results in the degradation of mitochondria via the autophagy-lysosome machinery. Autophagy is a cellular catabolic process in which key organelles, such as mitochondria, are transported to lysosomes for degradation [91]. PGC-1α can regulate transcription factor EB (TFEB) to mediate autophagy. The mTOR is another key autophagy regulator integrating amino acid starvation and increases of ROS to the autophagy pathway activities [92]. Autophagy is inhibited by the mTOR, while the negative regulator of mTOR (e.g., AMPK) promotes it. Both mTOR and AMPK can oppositely regulate unc-51-like kinase 1/2 (ULK1/2) activity by phosphorylation. In addition to activating ULK1/2, AMPK can promote autophagy through SIRT1. SIRT1 can deacetylate and activate several autophagy-related (Atg) proteins, such as Atg5, Atg7, and Atg8 [93]. Importantly, these regulatory pathways of autophagy are mainly activated upon ROS overproduction, NO deficiency, or nutrient deprivation [91,92]. Because mitochondria are a major source of ROS within most mammalian cells, these observations suggest that there are close interconnections among autophagy, mitochondrial ROS, NO production, and cell apoptosis vs. survival [94]. Given the essential role of endothelial dysfunction in the pathogenesis of CVD, disturbed autophagy in response to early-life nutritional insults in endothelial cells are likely to have significant contributions to cardiovascular programming.

Four lines of evidence suggest that the l-arginine–ADMA–NO pathway mediates nutrient sensing mechanisms to drive programming of CVD. First, recent reports showed that endothelial NOS is needed to couple AMPK activation to mammalian target of rapamycin complex 1 (mTORC1) [95], which is required for embryonic cardiovascular development and for postnatal maintenance of cardiac structure and function [96]. The second line of evidence consistent with an important role for l-arginine in nutrient sensing and fetal programming is that multiple catabolic fates enable l-arginine to have multi-faced functions in the development of the cardiovascular system [26]. In addition to NO, l-arginine is a nitrogenous precursor for synthesis of ornithine, polyamines, proline, glutamine, creatine, and agmatine. Many products of its catabolism are essential for a healthy pregnancy and fetal development. Indeed, many reports have demonstrated the beneficial effects of l-arginine supplementation in fetal outcome and CVD [23,26]. Third, there are studies that indicate that placental mTOR signaling regulates amino acid transporter and l-arginine regulates the mTOR signaling pathway [97,98]. These observations demonstrate a crosstalk between the l-arginine–NO pathway and the nutrient sensing signaling pathway in the placenta. Thus, placental nutrient sensing plays a critical role in modulating maternal-fetal resource allocation, affecting fetal programming and life-long CV health [99]. Fourth, our recent study showed that NO depletion in pregnancy induced programmed hypertension in adult offspring, which was associated with massive alterations in renal transcriptome [46]. By using next-generation sequencing (NGS) techniques, we observed that more than 2000 genes of renal transcriptome were altered by maternal *N^G^*-nitro-l-arginine-methyl ester (l-NAME) administration (a NOS inhibitor). Furthermore, our NGS data indicated that early redox imbalance programs a diverse range of pathways in later life, including the PPAR signaling pathway [46]. Additionally, we identified that several genes related to the nutrient sensing signaling pathway were significantly differential expressed, such as *Ppargcia* (encoding for the protein PGC-1α, fold change = 2.98), *Prkag3* (encoding for the γ3 chain of AMPK, fold change = 0.48), *Slc7a12* (encoding for amino acid transporter, y+ system, member 12, fold change = 3.66), and *Fabp4* (encoding for fatty acid binding protein 4, fold change = 2.73). These observations suggest a possible mechanism that early NO deficiency programs nutrient sensing signals, leading to the development of CVD in later life.

## 6. Reprograming Strategy via Targeting NO-ROS Balance and Nutrient Sensing Signaling

As we previously reviewed, numerous interventions have been reported to lower ADMA levels and restore NO-ROS balance in human and experimental studies, such as angiotensin-converting enzyme inhibitors, angiotensin receptor blockers, aliskiren, vitamin E, l-citrulline, melatonin, aminoguanidine, pioglitazone, probucol, farnesoid X receptor agonists, resveratrol, *N*-acetylcysteine, fenofibrate, folic acid, metformin, oral contraceptives, and α-lipoic acid [6,18,19,100,101]. However, to date, a specific ADMA-lowering agent remains inaccessible in clinical practice. Because PRMTs control ADMA production, whereas DDAHs and AGXT2 regulate its metabolism, the discovery of specific PRMT inhibitors, DDAHs, and AGXT2 activators might help in developing a therapeutic approach to lower ADMA in the near future.

Given that oxidative stress is a major mechanism contributing to cardiovascular programming, it is a reasonable assumption that antioxidant supplementation would be part of potential therapy strategies for CVD. However, currently the cardiovascular benefits of antioxidant have not yet been proven [102]. Because CVD is a multifactorial disorder, there remains a lack of data on which organ-specific redox-sensitive signals are responsible for oxidative stress-related programming processes and which developmental window (e.g., in utero or at the pre-weaning stage) is appropriate for reprogramming [16]. Therefore, the identification of specific antioxidants targeting restoration of NO–ROS imbalance in early life may constitute a potential intervention aimed at reprogramming the development of CVD in adulthood.

Targeting nutrient sensing signals is another potential reprogramming strategy to prevent the development of CVD. Several dietary phytonutrients have been reported to decrease the risk for CVD [103]. In addition to their antioxidant properties, such nature-derived phytonutrients, like quercetin, fisetin, genistein, epigallocatechins, and resveratrol have been shown to act as AMPK activators [104,105]. Additionally, several kinds of AMPK activators have been studied in CVD in adulthood [106], including metformin, thiazolidinediones (TZDs), polyphenols, ginsenoside, α-lipoic acid, and 5-aminoimidazole-4carboxamide riboside (AICAR). However, the ability of early interventions with AMPK activators to prevent the development of CVD in later life still await further elucidation. Next, practically all sirtuin activators have been described only for SIRT1 [107]. Resveratrol is a natural phytonutrient that activates SIRT1, and which may help in the prevention of fetal programming [49,108]. Despite other SIRT1 activators that are structurally unrelated to resveratrol having also been developed, their roles in preventing the developmental programming of CVD remain unclear. Moreover, increasing evidence has shown that all three PPARs are involved in the pathogenesis of CVD and its comorbidities, and their ligands may be considered for therapeutic use to prevent the development of CVD [86]. To date, however, only a few studies have explored the impact of early intervention by PPAR modulators to prevent cardiovascular programming, as reviewed previously [88]. There remains a long road ahead to establish the particular PPAR modulator, the right developmental window, and organ-specific effects, to prevent cardiovascular programming in different models of fetal programming.

## 7. Conclusions

Despite advances in therapy, CVD kills millions of people every year all over the world. The burden of CVD continues to increase globally, including many children who are at risk at an early age. Extensive experimental animal studies have shown that the interplay among the l-arginine–ADMA–NO pathway, oxidative stress, and nutrient sensing signals contributes to cardiovascular programming. Although major progress has been made in research on cardiovascular programming, many challenges still lie ahead. Future programming research should aim to bridge the translational gap between animal models and human therapeutics. Underlying the DOHaD concept, research into effective reprogramming strategies for cardiovascular programming that begin early in life will have a profound impact on economic burden of CVD over the next few decades.

## Figures and Tables

**Table 1:** Cardiovascular programming related to oxidative stress in animal models.

Animal Model	Species	Cardiovascular Phenotypes	Programming Mechanisms Related to Oxidative Stress	Age at Evaluation	Ref.
Maternal protein restriction	Rats	Hypertension, vascular dysfunction	↑ Oxidative stress, ↓ glutathione	12 wk	[43]
STZ-induced diabetes	Rats	Hypertension	↑ ADMA, ↓ NO	12 wk	[44]
Maternal l-NAME administration	Rats	Hypertension	↑ Oxidative stress	12 wk	[45,46]
Maternal suramin administration	Rats	Hypertension	↑ ADMA, ↓ NO, ↓ H_2_S	12 wk	[47]
Maternal caloric restriction	Rats	Hypertension	↑ ADMA, ↓ NO	12 wk	[48]
Prenatal hypoxia plus postnatal high-fat intake	Rats	Vulnerable to cardiac ischemia/reperfusion (I/R) injury	↑ Superoxide production	12 wk	[49]
Maternal ethanol consumption	Rats	Coronary tissue proliferation	↑ Lipid peroxidation	12 wk	[50]
Protein restricted diet	Rats	Hypertension	↑ ROS production and ↓ Antioxidant capacity in heart	14 wk	[51]
Prenatal dexamethasone exposure	Rats	Hypertension	↓ NO	16 wk	[52]
Prenatal dexamethasone exposure plus postnatal high-fat intake	Rats	Hypertension	↓ NO, ↑ Oxidative stress	16 wk	[53]
Maternal caloric restriction	Rats	Hypertension, vascular dysfunction	↑ Superoxide production	16 wk	[54,55]
Prenatal hypoxia exposure	Rats	Endothelial dysfunction	↑ Oxidative stress in aorta	16 wk	[56]
Prenatal LPS exposure	Rats	Hypertension, endothelial dysfunction	↓ NO, ↓ antioxidant enzyme expression	19 wk	[57]
Maternal high-fat intake	Rats	Hypertension	↑ Lipid peroxidation, ↓ NO	24 wk	[58]
Maternal nicotine exposure	Rats	Hypertension, vulnerable to cardiac I/R injury	↑ Arterial ROS production	8 mo	[59,60]
Maternal caloric restriction	Rats	Hypertension, cardiac damage	↑ Oxidative stress in heart	22 mo	[61]
STZ-induced diabetes	Mice	Myocardial ischemia/reperfusion injury	↑ Oxidative stress	8 wk	[62]
Maternal protein restriction	Mice	Vulnerable to vascular injury	↑ Oxidative stress	11 wk	[63]
Maternal protein restriction	Mice	Atherosclerosis	↑ Oxidative stress	6 mo	[64]
Prenatal 11β-HSD inhibition	Mice	Endothelial dysfunction	↑ Oxidative stress	6 mo	[65]
Maternal high-fat intake	Mice	Hypertension	↑ Arterial ROS production, ↓ NO	7.5 mo	[66]
Prenatal betamethasone exposure	Sheep	Hypertension	↑ ROS production, ↓ NO	18 mo	[67,68]

Studies tabulated according to species and age at evaluation. wk = week. mo = month. STZ = Streptozotocin. l-NAME = *N^G^*-nitro-l-arginine-methyl ester. LPS = Lipopolysaccharides. 11β-HSD = 11β-hydroxysteroid dehydrogenase. ROS = reactive oxygen species. ↑ = increased. ↓ = decreased.

## Abbreviations

ADMA	Asymmetric dimethylarginine
AGXT2	Alanine-glyoxylate aminotransferase 2
CAT	Cationic amino acid transporter
CVD	Cardiovascular disease
DDAH	Dimethylarginine dimethylaminohydrolase
DOHaD	Developmental origins of health and disease
l-NAME	*N^G^*-nitro-l-arginine-methyl ester
mTOR	Mammalian target of rapamycin
NOS	Nitric oxide synthase
OCT	Ornithine carbamoyltransferase
PGC-1α	PPARγ coactivator-1α
PPAR	Peroxisome proliferator-activated receptor
PRMT	Protein arginine methyltransferase
RAS	Renin-angiotensin system
SDMA	Symmetric dimethylarginine
SIRT	Silent information regulator transcript
STZ	Streptozotocin
